# Grain-Boundary-Induced Alignment of Block Copolymer Thin Films

**DOI:** 10.3390/nano10010103

**Published:** 2020-01-04

**Authors:** Steven Gottlieb, Marta Fernández-Regúlez, Matteo Lorenzoni, Laura Evangelio, Francesc Perez-Murano

**Affiliations:** Instituto de Microelectrónica de Barcelona (IMB-CNM, CSIC), Bellaterra, 08193 Barcelona, Spain; stgottl@googlemail.com (S.G.); marta.fernandez@imb-cnm.csic.es (M.F.-R.); matteo.lorenzoni@gmail.com (M.L.); lauraevangelioaraujo@gmail.com (L.E.)

**Keywords:** nanolithography, block copolymers, directed self-assembly, grain boundaries, AFM, mechanical removal

## Abstract

We present and discuss the capability of grain boundaries to induce order in block copolymer thin films between horizontally and vertically assembled block copolymer grains. The system we use as a proof of principle is a thermally annealed 23.4 nm full-pitch lamellar Polystyrene-block-polymethylmetacrylate (*PS-b-PMMA)* di-block copolymer. In this paper, grain-boundary-induced alignment is achieved by the mechanical removal of the neutral brush layer via atomic force microscopy (AFM). The concept is also confirmed by a mask-less e-beam direct writing process. An elongated grain of vertically aligned lamellae is trapped between two grains of horizontally aligned lamellae. This configuration leads to the formation of 90° twist grain boundaries. The features maintain their orientation on a characteristic length scale, which is described by the material’s correlation length *ξ*. As a result of an energy minimization process, the block copolymer domains in the vertically aligned grain orient perpendicularly to the grain boundary. The energy-minimizing feature is the grain boundary itself. The width of the manipulated area (e.g., the horizontally aligned grain) does not represent a critical process parameter.

## 1. Introduction

Block copolymers consist of two or more chemically different polymer chains covalently bonded together [1]. Driven by the repulsive force between chemically different molecules, the chains self-assemble in periodic structures, with characteristic size between few nanometers and few tens of nanometers. These periodic structures can be used as high resolution, bottom-up templates for nanofabrication processes, as for example bit patterned media for hard disk drives [2,3,4], finFETs [5], and contact holes [6].

A single-crystalline self-assembly morphology (e.g., perfect long-range order) cannot be achieved during the annealing of block copolymer thin films, despite of the fact that single-crystalline morphologies represent the minimal achievable free energy [7]. The formation of a rich variety of defects leads to polycrystalline morphologies and consequently represents limiting factor for the use of block copolymer self-assembly in many applications requiring a low defect density. As of now, this insufficiently low defect density is one of the main problems for the integration of block copolymer lithography in high volume manufacturing (HVM) processes [8].

Chemical and surface patterns defined by top-down lithography are usually used to introduce long-range order and placement accuracy in block copolymer thin films. These patterns are referred to as guiding patterns. The approach of directing the self-assembly of block copolymers by means of topographical patterns is referred to as graphoepitaxy. This principle has been successfully employed by a number of groups, as reported in different works in the last years [9,10,11,12,13,14]. Very low defect densities can be obtained when the width of the confining trench is exactly or close to an integer multiple of the natural pitch of the block copolymer [15,16,17]. A suitable guiding pattern design permits it to direct the self-assembly of block copolymers in device-oriented features [5,18], induce long-range order in sub-10 nm half-pitch block copolymers [19] and sub-5 nm half-pitch liquid crystals [20].

The chemoepitaxy approach relies on the introduction of chemical patterns into an underlying polymeric substrate. The orientation of block copolymers can be successfully controlled by the use of self-assembled monolayers [21] and crosslinked polymer mats [22] as underlayers. Common ways to manipulate the surface free energy of a Polystyrene-random-polymethylmetacrylate (*PS-r-PMMA)* brush layer is to combine a lithography step with the exposure to UV light [23] or to oxygen plasma [24]. The critical dimension of the guiding pattern features has to correspond to (n − 0.5) times the block copolymer pitch [25,26] with n being a positive integer.

In contrast to the well-explored approaches of graphoepitaxy and chemoepitaxy, there are only few works that study the use of grain boundaries to introduce order in block copolymer thin films. On the other side, the shape of grain boundaries in block copolymers and the energy minimization process associated to their formation in lamel lar block copolymers have been studied quite extensively for bulk materials, and reported in excellent experimental [27,28,29,30] and theoretical [31,32,33,34] works. A brief review of grain boundary morphologies in block copolymers can be found in Appendix A.1.

The few existing works on 90° twist grain boundaries in relation with block copolymer thin films include, for example, experiments with two chemically patterned plates [35], where the grain boundaries are formed parallel to the substrate, and with soft graphoepitaxy, where 90° twist grain boundaries have been observed for film thicknesses significantly larger than the guiding pattern feature height [36]. The ordering of block copolymers perpendicular to grain boundaries has recently also been identified as a metastable state in the chemoepitaxial alignment of block copolymers [37] and referred to as “stitch morphology”. Interestingly, in reference [38], the authors are capable of directing the self-assembly of block copolymers by the creation of structures under the formation of 90° tilt grain boundaries by mixing the pure block copolymer with a determined ratio of its constituent homopolymers, although it does not represent the energetically most favorable grain boundary morphology for pure block copolymer films [39]. Chemoepitaxy has been used to direct block copolymers into device-oriented features [40]. Despite of few isolated examples, grain boundaries have not been considered as order-inducing features in the directed self-assembly of block copolymers.

**Figure 1:**
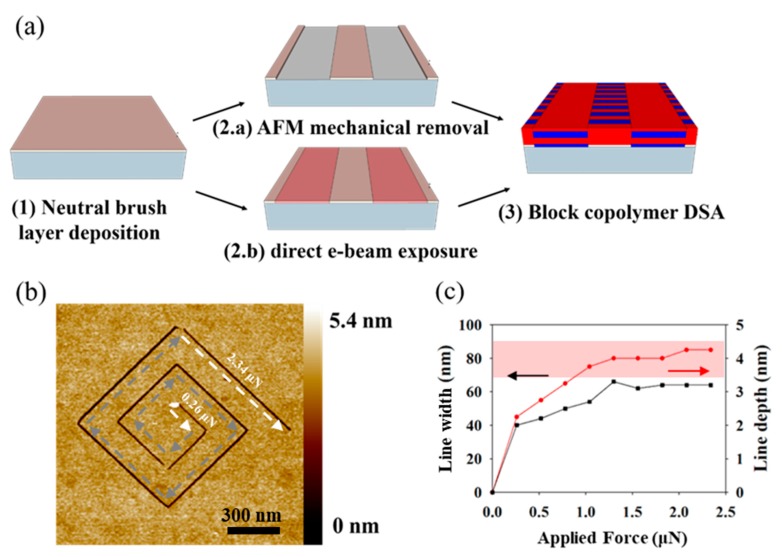
Grain boundary induced alignment principle and work-flow. (**a**) Work-flow to direct the self-assembly of block copolymers by grain-boundary-induced alignment; (**b**) Force-dependence of the mechanical brush removal while successively increasing the contact force from 0.26 μN to 2.34 μN from the inside to the outside of the spiral. (**c**) Linewidth (y-axis on left hand side) and line depth (y-axis on right hand side) as a function of the applied force extracted from the pattern shown in (**b**).

In this work, we trap one grain of vertically self-assembled block copolymers between two elongated grains of horizontally aligned block copolymers (see Figure 1a). With vertically self-assembled block copolymers we refer to areas where the lamellae are oriented perpendicular with respect to the substrate, while they are oriented parallel to the substrate in case of the horizontal self-assembly morphology. The driving force of the directed self-assembly in this set-up originates from 90° twist grain boundaries formed on both sides of the trapped vertically oriented grain. These naturally formed grain boundaries are the reason any other guiding pattern features are redundant.

We present grain-boundary-induced alignment (GBIA) as an interesting complementary technique to direct the self-assembly of block copolymers, because it represents a versatile method to align the material on lengths up to its correlation length *ξ*. The correlation length *ξ* characterizes the length range where the self-assembly of block copolymers can be successfully directed by grain-boundary-induced alignment. The parameter *ξ* is inversely proportional to the defect density in the film [41] in fingerprint pattern. Due to the absence of guiding patterns in a stricter sense, grain-boundary-induced alignment does not increase the mean grain size of the material, but rather orients the grains perpendicular to the formed grain boundaries.

## 2. Results

The fabrication of aligned block copolymer patterns via (GBIA) has been realized using two different nano-patterning methods: (i) Atomic force microscopy (AFM) mechanical removal (m-AFM) and (ii) direct e-beam exposure of the underlying neutral brush layer. In block copolymer lithography, brush layers are commonly used to control the free energy between the substrate and the block copolymer thin film. Most commonly, the brush layer is a random copolymer consisting of the same two components as the used block copolymer. In case the random copolymer (here: Polystyrene-random-polymethylmetacrylate (*PS-r-PMMA*)) consists of the same or similar volume fraction as the block copolymer (here: Polystyrene-block-polymethylmetacrylate (*PS-b-PMMA*)), the surface is wetted by both two block copolymer components. A brush layer that favors vertical self-assembly in block copolymers is referred to as neutral brush. In the specific case of a lamellar block copolymer as used in this work, a neutral brush leads to vertically standing block copolymer domains. The two workflow variations are presented in the two following sub-sections and they are depicted in Figure 1a. As we will explain in the following sections, the selective treatment of the brush layer is used to manipulate the interface energy between the block copolymer and the substrate in such a way that we are capable of changing the self-assembly mode of the block copolymer from vertical to horizontal.

### 2.1. Alignment Induced via Mechanical AFM (m-AFM)

As depicted in Figure 1a, sketch (2.a), the fabrication of contact pads in the m-AFM approach relies on the mechanical removal of the neutral brush layer using an AFM tip. The AFM height image depicted in Figure 1b represents a force test to determine the most suitable contact force for the mechanical removal of the neutral brush layer. Here, the contact force has been increased from 0.26 μN to 2.34 μN in steps of 0.26 μN each segment, where the lowest contact force is applied for the innermost segment of the spiral (see Figure 1b). Based on this experiment, we determine 1.04 μN as the minimum required contact force to ensure the complete removal of the neutral brush layer down to the substrate (see Figure 1c), because we observe that both line width and line depth enter in a plateau regime where they are no longer a function of the applied force, i.e., further increasing the contact force further does not lead to a larger indentation depth.

**Figure 2:**
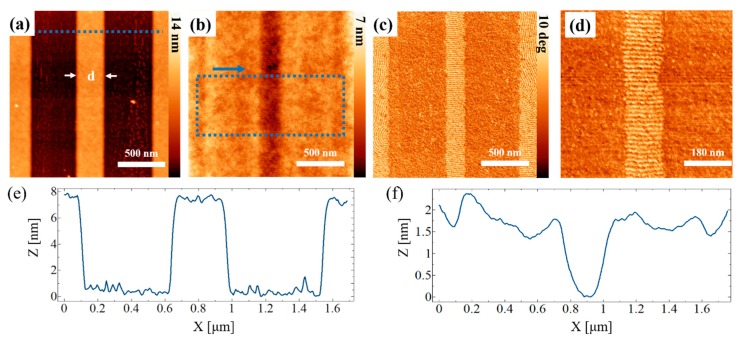
Results of grain-boundary-induced alignment via atomic force microscopy (AFM) mechanical removal. (**a**) AFM height image of guiding pattern with two areas treated by AFM mechanical removal (m-AFM) without brush layer (corresponds to sketch 2.a in Figure 1); (**b**) AFM height image of directed self-assembly (DSA) on area depicted in (**a**); (**c**) AFM phase image taken simultaneously to image (**b**), showing the self-assembly in horizontal (wide stripes) and vertical (narrow stripes) lamellae; (**d**) Zoom-in the central stripe of the AFM phase image of (**c**); (**e**) single line profile along blue dashed line in (**a**); (**f**) average height profile (i.e., average of all line scans) as indicated by the box in (**b**).

For the first set of experiments, we have removed the neutral brush layer in areas of 500 nm × 5 μm (Figure 2a). The width of the areas has been chosen so that the resulting structures have a reasonable size for a later characterization. The mechanical removal uncovers the underlying silicon substrate, which is preferentially wetted by *PMMA.* During the self-assembly process, when the block copolymer is heated above its glass transition temperature, the formation of horizontal lamellae is induced in these areas.For block copolymer film thicknesses in the range of L_0_, (where L_0_ is the natural full pitch of the lamellar block copolymer), the top material is thus *PMMA*. On the other hand, the top-layer consists of *PS* when the film thickness is in the range of 1.5 *L_0_*.

As the m-AFM technique is physically displaces the random polymer brush, pile-ups are formed adjacent to the borders of the modified areas. In order to remove pile-ups of removed random copolymer and to be capable of analyzing the sample appropriately via AFM, the sample undergoes a Propylene glycol methyl ether acetate (*PGMEA)* rinsing step before taking the AFM height image in Figure 2a. The distance *d* between the two modified zones, as depicted in Figure 2a, is 300 nm. The height step of approximately 7 nm corresponds to the height of the random copolymer layer, which has been entirely removed in the two recessed, modified areas. The two recessed areas is what we refer to as guiding pattern for this application. The result of the directed self-assembly on these guiding patterns is depicted in an AFM height image (Figure 2b) and phase image (Figure 2c). Profiles revealing the sample topography perpendicular to the modified areas before and after the directed self-assembly step are presented in Figure 2e,f. The vertically aligned block copolymer structures have a notably shallower (and inversed) topography of around 2 nm. The block copolymer in the trapped grain adapts its thickness to the height of the adjacent horizontally self-assembled grains. Additional information concerning the analysis of effect is presented in Appendix A.2.

### 2.2. Alignment Induced via Electron Beam Direct Exposure

**Figure 3:**
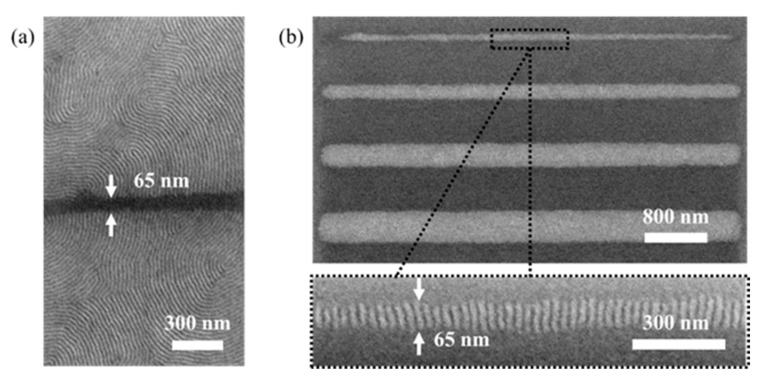
Results of grain-boundary-induced alignment obtained by electron beam direct writing. (**a**) Horizontal self-assembly on a 65 nm wide stripe; (**b**) vertical self-assembly (e.g., grain-boundary-induced alignment) on stripes of different widths with a minimum of 65 nm.

The second case is the surface modification by an exposure to an electron beam, where we make use of the modification of the neutral brush layer due to interaction with charged particles. As explained by Evangelio et al. [26], high electron beam exposure doses (e.g., 256 mC/cm^2^) cause a change in the surface free energy of *PS-r-PMMA* and convert the nominally neutral brush layer into a brush layer that is preferentially wetted by *PMMA.* As explained before, this effect, in turn leads to horizontal self-assembly similar to the one observed after the removal of the neutral brush layer, but dispensing with the physical removal of material. Figure 3 shows the results of self-assembly in the direct vicinity of a 65 nm wide line (Figure 3a) and a 65 nm wide pristine area Figure 3b. The orientation of the block copolymer perpendicular to the grain boundary is explained by the energy minimization inside the grain boundary and occurs as a direct consequence of the formation of 90° twist grain boundaries.

### 2.3. Results of Pattern Transfer of Directed Features into Silicon

**Figure 4:**
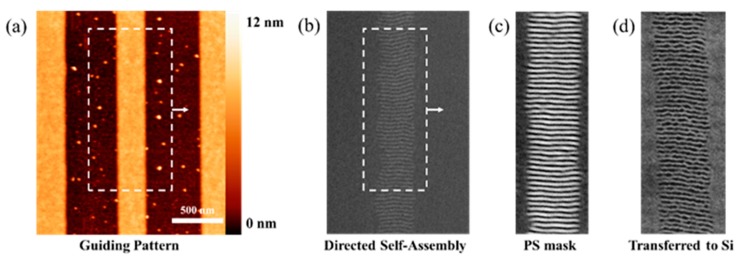
Micrographs of different fabrication stages in the pattern transfer process. (**a**) AFM image of m-AFM treated area for grain-boundary-induced alignment; (**b**) DSA of block copolymers on the presented guiding pattern, while the excerpt corresponds to area surrounded by dashed line in (**a**); (**c**) *PS* etch mask after selective removal of *PMMA* via Reactive Ion Etching (RIE) (area corresponds to white dashed line in (**b**)); (**d**) pattern transferred into silicon.

To make use of grain-boundary-induced alignment for lithography applications, it is important to be capable of transferring the defined structures into silicon. As it is depicted in Figure 4, the pattern transfer process consists of two steps. After the guiding pattern fabrication (see Figure 4a) and the self-assembly (see Figure 4b), it is necessary to remove the *PMMA*, so that the remaining *PS* can serve as an etch mask. This process step is displayed in Figure 4c, where the bright lines correspond to *PS* features and the dark lines correspond to the voids created by removed *PMMA*. The *PMMA* removal can effectively be done using various oxygen-containing gas mixtures, such as *Ar*/*O*_2_ or *CHF*_3_/*O*_2_ (see Materials and Methods part of this paper for more information). *PS*, in turn, is relatively more inert than PMMA towards oxygen-containing plasma etch processes, enabling an etch selectivity around 3.

Secondly, we use the remaining *PS* template as an etch mask to transfer the defined features into silicon. The result of the pattern transfer of few nm depth into silicon is depicted in Figure 4d. A great advantage for the fabrication of devices with this technique is that the horizontally aligned block copolymers serve as an etch mask and preclude the chemical attack of Si in this area. If the process is conducted on a Silicon-on-Insulator (SOI) wafer, these areas can subsequently be used as electrical contacts, because the silicon below remains intact. This work-flow may represent a simple method for the fabrication of dense nanowire arrays for nanoelectronic devices.

## 3. Discussion

### 3.1. Surface Energy Modification by m-AFM

As the surface energy modification of a polymeric brush layer by direct e-beam exposure has been presented elsewhere [26], in this section we will focus on the surface energy modification induced by the m-AFM step.

**Figure 5:**
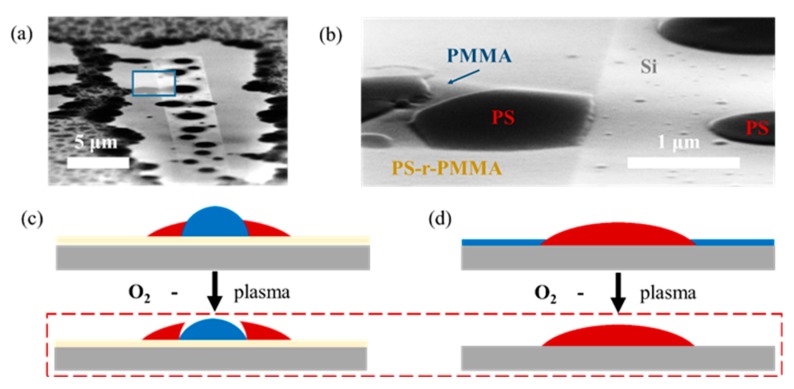
Qualitative analysis of *PS/PMMA* blends on neutral surfaces and on surfaces modified by m-AFM. (**a**) Overview SEM image of *PS/PMMA* blend in two modified areas (stripes) and their direct pristine vicinity covered with neutral brush layer, (**b**) close-up of the part of (**a**) indicated by the box drawn in light blue, (**c**) explanatory sketch of the architecture of the droplet sketched on the left side of (**b**) before and after the oxygen plasma treatment, (**d**) explanatory sketch of surface on the right side of image (**b**).

The analysis of the wetting behavior of a homopolymer blend consisting of the components of the block copolymer can serve as a qualitative test for the surface energy in micrometric areas [26,42]. Here, we use this technique to qualitatively understand the surface energy by observing the behavior of a *PS*/*PMMA* blend in the modified and in the non-modified area. Phase separation of *PS* and *PMMA* is induced by the annealing of the film at 230 °C for few minutes. To be capable of distinguishing the two polymers more easily in the SEM images, we subjected the sample to an 18 s oxygen plasma treatment at 500 W source power. Due to the higher etch resistivity of *PS* with respect to *PMMA*, we expect the *PMMA* droplets to be recessed in height. The results are depicted in Figure 5. Figure 5a shows the behavior of the polymer blend in the close vicinity of a 5 μm × 50 μm stripes, where the neutral brush layer has been removed by means of m-AFM. In Figure 5b we observe a behavioral difference of the polymer blend between the modified area and the pristine area in more detail.

The phase-separated *PS*/*PMMA* droplet in the pristine area of the sample in Figure 5b is sketched in Figure 5c and provides valuable insight in the behavior of polymer blends on neutral surfaces. The slightly recessed part of the droplet corresponds to a *PMMA* droplet inside a *PS* droplet due to the *O*_2_ plasma treatment. This indicates that both polymers have very similar interface energies with respect to the neutral brush layer. The surface energy of *PMMA* (with respect to the air) is slightly higher than of *PS* [43], which justifies that the *PMMA* droplet is inside the *PS* droplet and not vice versa. A similar behavior has been presented before for the investigation of chemical guiding patterns [42,44].

In contrast, we do not observe these characteristic twin-droplets in the modified areas. This behavior is explained by a homogenous coverage of the silicon by *PMMA*, because the interface energy between the activated silicon and *PMMA* is significantly lower than between silicon and *PS*. The *PS* thus minimizes its surface energy through the formation of droplets surrounded by a continuous *PMMA* layer. This concept is sketched in Figure 5d. It is concluded that the surface energy is efficiently modified when the neutral brush layer is removed by m-AFM.

### 3.2. Limits of Grain-Boundary-Induced Alignment

An important feature of the ordering of block copolymers via grain-boundary-induced alignment is that it does not require the fabrication of a high-resolution guiding pattern. This comes, however, at the expense of the fact that the maximum alignment length is limited by the correlation length, *ξ,* of the block copolymer. To estimate a reasonable maximum distance *d* between the two horizontally aligned grains, we have to know the grain size distribution of the block copolymer finger print after self-assembly.

**Figure 6:**
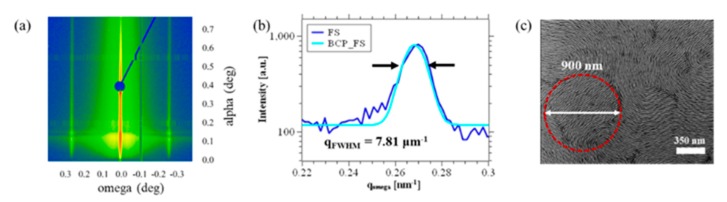
Estimating the limits of the directed self-assembly by grain-boundary-induced alignment. (**a**) Grazing-incidence small-angle X-ray scattering (GISAXS) pattern of randomly assembled block copolymer features; (**b**) estimation of the full-width at half maximum (FWHM) of the block copolymer grating truncation rod (GTR) to estimate the mean correlation length ξ of the sample; (**c**) SEM image of finger-print pattern with a circle representing the mean grain size as determined by the analysis of the GISAXS pattern.

In Figure 6 we present an estimation of the block copolymer correlation length based on the grazing incidence small angle X-ray scattering (GISAXS) pattern depicted in Figure 6a, similar to how it has already been discussed elsewhere (for example in [44,45]). Moreover, we present an analysis of the width of the first order grating truncation rod (GTR) in Figure 6b, and an SEM image indicating the estimated mean grain size in Figure 6c.

It is well-known that block copolymer self-assembly is a process based on grain nucleation and subsequent growth, also referred to as coarsening [46]. Characterization techniques like small-angle X-ray scattering (SAXS), depolarized light scattering (DPLS) [47], and grazing-incidence small-angle X-ray scattering (GISAXS) [44] are capable of providing mean values [48] for the grain sizes in block copolymers (e.g., correlation length). Nevertheless, the mean value does not, a priori, contain information about the distribution of the grain sizes. However, the experimental analysis of the grain size in the self-assembly of horizontally aligned diblock copolymers by AFM has been demonstrated to be in excellent agreement with a log-normal distribution function [49]:

(1) $$f\left(\ln\left(\xi \right)\right)=\frac{1}{\sqrt{2\pi }*\ln\left(\sigma \right)}*exp\left\{-\frac{{\left(\ln\left(\xi \right)-\ln\left(\mu \right)\right)}^{2}}{2*ln^{2}\left(\sigma \right)}\right\}$$

with *σ* being the geometric standard deviation and *μ* being the number-based geometric mean, equivalent to the mode diameter of the grain.

**Figure 7:**
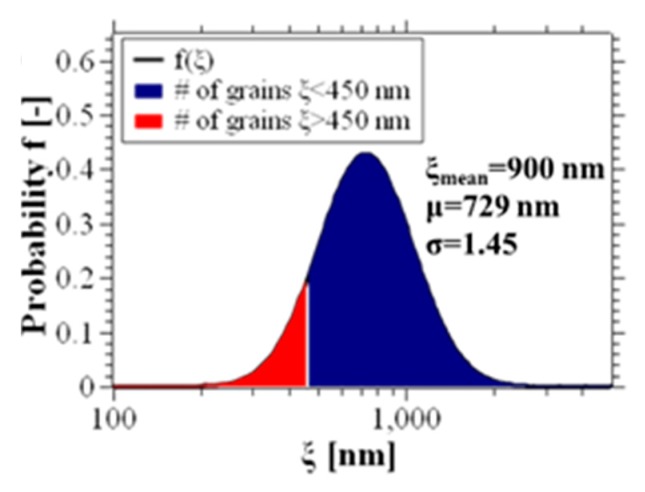
Grain size analysis in block copolymer thin films. Grain size distribution for the block copolymer in free surface for *ξ_mean_* = 900 nm

The authors of [49] suggest that the block copolymer grain size can be described by a Smoluchowski coagulation function, for which *σ* = 1.45; usually used for systems where particle trajectories are controlled by Brownian motion. If the correlation length, *ξ_GISAXS_* as determined by the GISAXS line width analysis, corresponds to the mean correlation length of all grains (in this case *ξ* = 900 nm), we can estimate that the grain size distribution f(ln(ξ)) of our sample is:

(2) $$f\left(\ln\left(\xi \right)\right)=\frac{1}{\sqrt{2\pi }*\ln\left(1.45\right)}*exp\left\{-\frac{{\left(\ln\left(\xi \right)-\ln\left(731.7\right)\right)}^{2}}{2*ln^{2}\left(1.45\right)}\right\}$$

(plotted in Figure 7a). The number 731.7 is selected to obtain the experimentally observed value for $\xi_{mean}$:

(3) $$\xi_{mean}=\;\frac{\int_{0}^{\infty }\xi *f\left(\ln\left(\xi \right)\right)d\xi }{\int_{0}^{\infty }f\left(\ln\left(\xi \right)\right)d\xi }=900\;\mathrm{nm}$$

Knowing the approximate grain size distribution of our block copolymer in free surface f(ln(ξ)), we can estimate the probability *p* [%], that a grain of the sample is smaller than a determined value *ξ_0_*:

(4) $$p\left(\xi_{0}\right)=\;100*\frac{\int_{0}^{\xi_{0}}f\left(\ln\left(\xi \right)\right)d\xi }{\int_{0}^{\infty }f\left(\ln\left(\xi \right)\right)d\xi }\left[\%\right]$$

The particle distribution function in Figure 7a is divided in a red and a blue area. The surface area of the red part divided by the total area represents the probability *p*(*ξ_0_*) that an areal unit forms part of a grain smaller than *ξ_0_* = 450 nm, which in this case is 4.8%. Accordingly, 0.3% of the total area is occupied by grains with the size of 300 nm and merely 7 × 10^−10^% of the area is occupied by grains smaller than 65 nm.

The self-assembly in structures like the ones we fabricate in this work is considered to be successful if there is no defect on the entire length of the grain. We can estimate the probability $p_{\bar{d}}$ of this event by estimating the probability that all the grains along the grain boundary with the length *l* are at least as large as the distance *d* between the two grain boundaries, which is given by the term:

(5) pd¯=p(ξ0=d)ld

**Figure 8:**
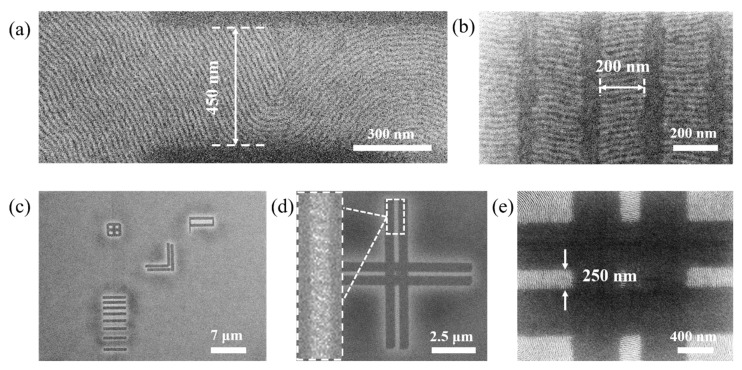
Alternative structures fabricated by grain-boundary-induced alignment. (**a**) Defective self-assembly due to a too large distance between the two grain boundaries; (**b**) self-assembly of multiple arrays of parallel lamella; (**c**) arbitrary shapes fabricated by mechanical AFM removal; (**d**,**e**) double-bar cross defined by mechanical AFM removal.

In the table presented in Table 1 we present the probability to fabricate a *l* = 5 μm long array without defects for three different lengths of *d*. The values for *d* we worked with in this table are process parameters used in this section. In particular, we have presented the grain boundary-induced alignment with *d* = 300 nm in Figure 2 fabricated by m-AFM and *d =* 65 nm in Figure 3 by e-beam direct writing and we observe no defects in these structures—just as predicted by the presented estimation. The fact that a $p_{\bar{d}}\left(d=450\;\mathrm{nm}\right)$ yields less than 60% indicates that the probability to find defects in such a structure rather high. An example of a structure with *d =* 450 nm is depicted in Figure 8a and we observe the formation of defects.

Based on the presented analysis, the self-assembly of block copolymers by grain-boundary-induced alignment mainly depends on the correlation length *ξ* of the block copolymer (material parameter), and on the distance *d* between the two horizontally aligned grains and the length *l* of the horizontally aligned grains (process parameters). For this reason, it is important to understand the mechanisms of self-assembly and defect-annihilation in detail, which has already been subject to a number of works [41,44,50,51].

The rate at which a block copolymer eliminates defects in the course of the self-assembly process is determined by the energy barrier that has to be overcome in order to annihilate the defect [52]. Here, a smaller energy barrier indicates a faster defect removal mechanism which is expressed by a higher degree of order in the block copolymer. In [52] the authors demonstrate that the energy barrier for the defect removal is inversely proportional to *χ*N. A direct consequence of that is that the correlation length *ξ* of block copolymers is larger for small-pitch materials (e.g., materials with small *χ*N). This means that an inherent property of grain-boundary-induced alignment is the decreasing number of defects for smaller-pitch materials. Additionally, the influence of different fractions of homopolymer in the block copolymer on both grain boundary energy and correlation length is investigated and presented in Appendix A.3.

### 3.3. Fabrication of Patterns of Arbitrary Geometry

The fabrication of patterns to direct the self-assembly of block copolymers by grain-boundary-induced alignment by m-AFM is not limited to trapping one single grain, which has been the only structure that we have discussed until now. In Figure 8b–e, we present alternative structures fabricated by grain-boundary-induced alignment, such as defect-free array of nanowires of 200 nm in length and a pitch of 250 nm in Figure 8b. In Figure 8c we show a number of geometric shapes that have been fabricated to demonstrate the versatility of the technique. A double-lined cross of 10 μm × 10 μm with a line width of 500 nm and 250 nm spaces between the lines is depicted in Figure 8d,e. The in-set of Figure 8d shows the Moiré pattern between the two branches of the cross, which indicates the high order of the block copolymer without actually having sufficient measurement points to resolve single block copolymer domains. Moiré patterns are interference patterns [53] that occur when a periodic lattice (for example a directed block copolymer pattern) is measured with an imaging technique whose sampling step size is below the step size of the lattice that is supposed to be measured. The existence of the Moiré pattern hence can be understood as a proof for the successfully directed self-assembly of the block copolymers between the two branches of the cross. To verify this thesis, we show a close-up of the center of the fabricated cross in Figure 8e, where we doubtlessly see the directed self-assembly of block copolymers is successful in each one of the four trapped grains.

## 4. Materials and Methods

### 4.1. Substrate

The substrate are chips cut from a <100> Si wafer. Native oxide is not removed. After cleaning the substrate in isopropyl alcohol and acetone, the substrate undergoes an oxygen plasma treatment for 600 s at 500 W.

### 4.2. Neutral Brush Layer Deposition

The neutral brush layer consists of grafted *PS-r-PMMA* polymer chains (58 wt% *PS*, 42 wt% *PMMA*, M_p_ = 7.9 kg/mol and polydispersity index 1.85) yielding a film thickness of 6.5. To deposit the neutral brush layer, 1.5 wt% of *PS-r-PMMA* is dissolved in *PGMEA* and the solution is spin-coated to the silicon wafer for 30 s at 5000 rpm. To graft the molecules to the surface, the chip is annealed at 230 °C in a nitrogen atmosphere for 300 s. The non-grafted molecules are removed by rinsing the sample in *PGMEA.* All the polymer materials have been supplied by Arkema (Colombes, France).

### 4.3. Guiding Pattern Fabrication

In this work we use two different methods to fabricate the guiding patterns. The guiding pattern fabrication by AFM consists of removing the neutral brush layer in two elongated rectangular areas of 500 nm × 5 μm separated by a distance *d* in the range of few hundreds of nanometers. The brush removal is done by mechanical AFM using the contact mode of a Dimension Icon/Nanoscope V AFM (Bruker Corporation, Billerica, MA, USA). The tips used in these experiments (*OTESPA*, also from Bruler, *Si*-tip with nominal spring constant 42 N/m) have a nominal apex radius of 7 nm in the unused state. We estimate the contact force for the probed deflection set-points to be between 0.26 μN and 2.34 μN. The AFM height image shown in Figure 1b shows the efficiency of the polymeric brush layer removal as a function of the contact force. The contact force has been increased by 0.26 μN for each segment of the spiral going from the inside to the outside. The fabrication of the structures presented in this article has been conducted with nominal contact force of 1.04 μN, because this condition represents the minimum required force to remove the polymer film down to the substrate.

The second approach to fabricate the guiding patterns is based on the modification of the brush layer by direct e-beam exposure. The exposure has been performed in a *RAITH 150 (TWO)* electron lithography tool (Raith Gmbh, Dortmund, Germany) with a nominal beam diameter of 2 nm. We expose lines of 50 μm in length and between 65 nm and 500 nm in width, with separations between 500 nm and 65 nm. We applied the same conditions as used in reference [26]: electron beam with 20 kV acceleration voltage; sample current of 330 pA; exposure dose of 256 mC/cm^2^.

### 4.4. Block Copolymer Deposition

The diblock copolymer is a *PS-b-PMMA* (Arkema (Colombes, France) consisting of 42 wt% *PS* and 58 wt% *PMMA*. Upon self-assembly, this material forms 23.4 nm full-pitch lamellar features. Its polydispersity index *PDI* is 1.1, and the molecular weight is 42.3 kg/mol. A 1.7 wt% solution of the polymer in *PGMEA* is deposited by spin coating for 30 s at 2500 rpm and subsequently annealed for 600 s in a *N*_2_ atmosphere at 230 °C. This process yields a film thickness of 24 nm in free surface. 

### 4.5. Pattern Transfer

The pattern transfer consists of two steps. At first, the *PMMA* block is removed in a selective dry etching step in an Alcatel AMS 110 DE ICP-RIE. (Inductively coupled plasma reactive ion etching). We used etching conditions similar to those previously successfully developed and used by the authors of reference [54]: a gas mixture of 200 sccm *Ar* and 10 sccm *O*_2_ with at 200 W source power and 5 W substrate power. The etch selectivity of *PS* with respect to *PMMA* in this process is 1:3 with *PS* being the more resistant material. The etching time for this step is 21 s. For the subsequent *Si* etching we use the same RIE tool with a plasma power of 1200 W and a substrate power of 10 W for 12 s. The used gases are 30 sccm *SF_6_* and 25 sccm *C_4_F_8_*. We employ an etch process in which both gases are injected in the reaction chamber in a non-pulsed way.

### 4.6. GISAXS Measurements

GISAXS measurements of a representative sample have been conducted at the P03 Micro- and Nanofocus X-Ray Scattering Beamline at PETRA III in Hamburg [55]. The sample-detector distance was 5800 mm and the radiation wavelength 0.107 nm. The incidence angle of the beam was 0.4°. The detector that has been used for these experiments is a PILATUS 300k pixel detector (ECTRIS Ltd., Baden-Daettwil, Switzerland) with a readout time *t* < 3 ms and a pixel size of 172 μm.

## 5. Conclusions

We have shown that the directed self-assembly of block copolymers by grain boundary induced alignment is possible either by the controlled removal of an intermediate polymeric brush layer or its local surface modification. We have shown this concept using both a probe-based mechanical removal approach and an electron-beam direct writing approach.

The driving force of the self-assembly is the energy minimization process in the grain boundary between horizontally and vertically assembled block copolymers. This specific grain boundary is referred to as 90° twist grain boundary, where the *PS*/*PMMA* surface is reduced to a first Scherk surface, which mathematically represents a minimal surface. Because this approach does not require a guiding pattern along the self-assembly direction, the correlation length *ξ* is the limiting factor for the defect-free alignment.

For this reason, grain-boundary-induced alignment represents a particularly interesting alternative for early stage testing of new high-*χ*, low-pitch block copolymers with large correlation lengths. A recent report on the self-assembly of sub-5 nm liquid crystals (with a remarkably high degree of intrinsic order) has shown that the alignment of very small features by the mean of graphoepitaxy may be perturbed by large guiding pattern roughness [20]. Grain-boundary-induced alignment offers one possible solution for upcoming challenges that may be encountered by graphoepitaxy (and also chemoepitaxy), which is the difficulty to provide reliable guiding patterns for very small pitch self-assembling materials. Grain-boundary-induced alignment, in turn, favors the directed self-assembly of materials with small pitches due to their large correlation length.

On the down-side, we have to remark that the limited placement accuracy of the block copolymer features may represent an issue that requires improvement. This study presents a straight forward way to direct the self-assembly of block copolymers by taking advantage of the grain boundaries created between two differently oriented areas of block copolymers.

## Figures and Tables

**Table 1:** Results for the probability of defect free self-assembly between two 5 μm grain boundaries for three characteristic distances d between the grains. Average correlation length $\xi_{mean}$ is 900 nm.

Distance *d* between Two Grains	Probability $p_{\bar{d}}$ of Defect-Free Self-Assembly
65 nm	99.99999%
300 nm	95.15%
450 nm	57.89%

**Figure A1:**
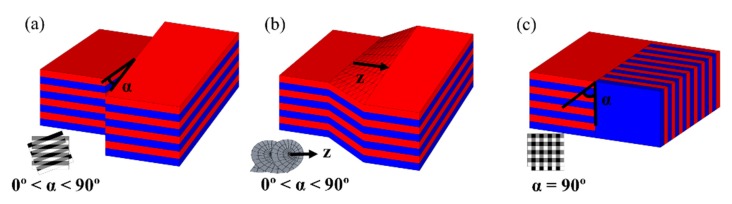
Different types of twist grain boundaries and their corresponding inter-material dividing surface (IMDS). (**a**) Two grains of lamellar block copolymers under a twist angle α ≪ 90°; the grey areas of the inset represent the IMDS reconstructed in a Scherk surface; (**b**) two grains of lamellar block copolymers under a twist angle α ≪ 90°, the inset represent the IMDS reconstructed in a helicoid shape; (**c**) two grains of lamellar block copolymers under a twist angle of 90°, the grey areas of the inset represent the IMDS reconstructed in a Scherk surface.

**Figure A2:**
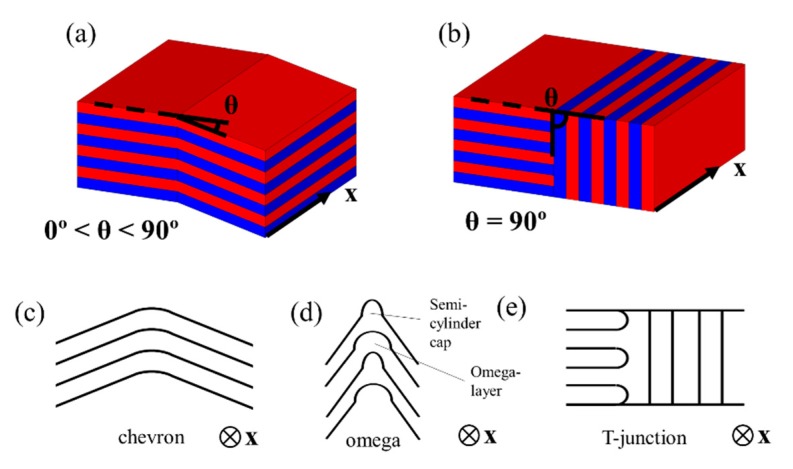
Overview of tilt grain boundary morphologies. (**a**) Two grains of lamellar block copolymer tilted under an angle well below 90°, (**b**):two grains of lamellar block copolymer tilted under an angle of 90°, (**c**) 2D-scheme of the IMDS of a low-tilt-angle chevron grain boundary, (**d**) 2D-scheme of the IMDS of an omega grain boundary, (**e**) 2D-scheme of the IMDS of a pure T-junction as observed in 90° tilt grain boundaries.

**Figure A3:**
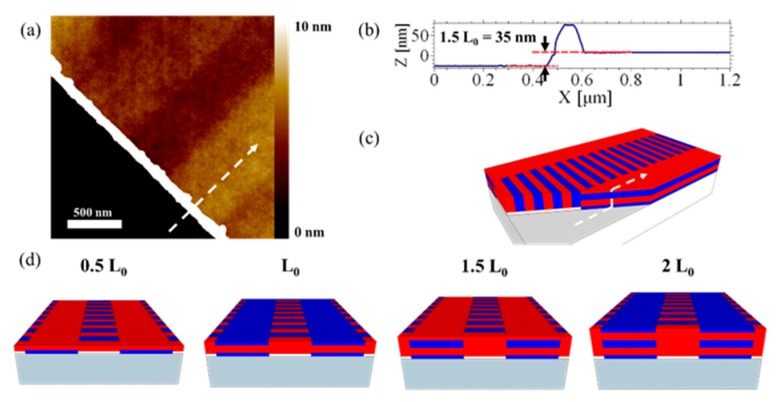
Measuring the height step between substrate and horizontally aligned block copolymer lamellae. (**a**) AFM height image of prepared sample; (**b**) single line profile indicated by white dashed line in (**a**); (**c**) 3D sketch of the measurement presented in (**a**); (**d**) possible self-assembly morphologies with height quantized to from 0.5 L_0_–2 L_0_ in steps of 0.5 L_0_.

**Figure A4:**
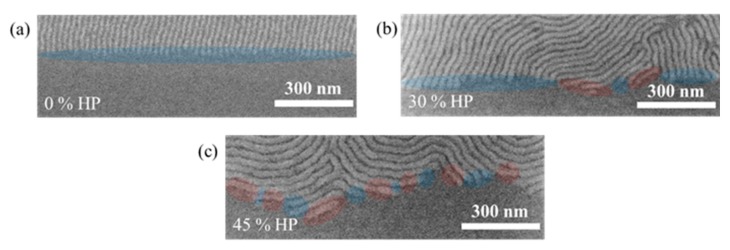
Grain boundary evolution as function of homopolymer content in the ternary blend. (**a**) 0% homopolymer, (**b**) 30%, (**c**) 45%; tilt grain boundaries and the blue areas are marked in red and twist grain boundaries are marked in blue.

## Appendix A

### Appendix A.1. Overview of Grain Boundary Morphologies in Block Copolymers

Two relevant types of grain boundaries are the twist grain boundary at one hand (see Figure A1) and the tilt grain boundary on the other hand (see Figure A2). As for lamellar block copolymers, the grain boundary free energy is a function of the area of the intermaterial dividing surface (IMDS) and the stretching and the compression of molecules in the close vicinity of the grain boundary. The material usually tries to reduce the area of the IMDS by stretching and compressing adjacent chains.

For twist angles *α* < 15° we observe two different reconstruction moduli, which are called helicoid interphase and Scherk first surface [32,34]. The two morphologies coexist for low angles because of their similar grain boundary energies (see Figure A1a,b). The major difference between these two morphologies is the shape of the IMDS. There is no major reconstruction mechanism in the formation of helicoid grain boundaries (see shape of the respective IMDS in inset of Figure A1b)). In the second case, the first Scherk surface, the IMDS is reconstructed to a distorted chess board pattern, as described in [31]. The IMDS in these structures can be mathematically described as a first Scherk surface, which forms part of the family of minimal surfaces [56]. The insets of Figure A1a,c represent a scheme of the situation inside the grain boundary. Here, white and black areas represent A-A-block interfaces and B-B-block interfaces, respectively. The actual IMDS is represented by the grey areas, which represent A-B-block interfaces. The reconstruction of twist grain boundaries in first Scherk surfaces has been observed experimentally [27,29]. For better understanding, we have plotted a scheme of a first Scherk surface as it occurs in 90° twist grain boundaries in Figure A1d. For large twist angles (*α* ≫ 15°), the formation of first Scherk surfaces is energetically favorable when compared to helicoid grain boundaries. This is at one hand because the helicoid grain boundary massively compresses the lamellae (and therefore the chains) at large angles. On the other hand, the first Scherk surface is by definition a minimal surface that minimizes the IMDS at large twist angles of *α =* 90°.

Tilt grain boundaries represent another grain boundary family commonly observed in bulk block copolymer samples. Schemes of two grains under a low tilt angle *θ* ≪ 90° and under a tilt angle *θ =* 90° are represented in Figure A2a,b. Low angle tilt grain boundaries are sub-divided in so-called chevron tilt grain boundaries and omega tilt grain boundaries. While the chevron tilt grain boundary is rather prominent at low tilt angles, the omega grain boundary tends to be the result of energy minimization at larger tilt angles [33]. As we can tell from the two sketches depicted in Figure A2c,d, both morphologies are similar in appearance. The sequence of the omega-layers and semicylinder caps [33] in omega tilt grain boundaries efficiently minimizes the grain boundary energy at higher angles. When the tilt angle increases even more and *θ* approaches 90° (see Figure A2b), the most efficient manner to minimize the free energy of the system is to interrupt the continuity of one of the species and reorganize them in semicylinder caps as shown in Figure A2e.

90° tilt and 90° twist grain boundary structures, as sketched in Figure A1c and Figure A2b, may form in block copolymer thin films, when a vertically oriented grain is adjacent to a horizontally oriented grain. This situation may occur wherever the surface energy changes abruptly from a neutral surface to a highly preferentially wetted surface. Taking into consideration that the block copolymer features on neutral surfaces are upright standing lamellae, the only two structures that may be formed in such grain boundaries are the 90° tilt grain boundary and the 90° twist grain boundary. In reference [36] the authors argue that the free energy of 90° twist grain boundaries is around half of the free energy of 90° tilt grain boundaries, which heavily benefits the formation of twist grain boundaries. This calculation is moreover supported by the fact that 90° twist grain boundaries, and the resulting tendency to form Scherk’s first surface IMDSs, are also observed in the terraced self-assembly horizontally aligned block copolymers on preferentially wetting substrates [57].

### Appendix A.2. Fixed-Height Self-Assembly

Transferring the created block copolymer patterns into the underlying substrate is a key step that converts block copolymer lithography into a purposeful technique for semiconductor manufacturing. Here, the pattern transfer process is particularly challenging, because polystyrene and poly(methyl methacrylate) are chemically similar. Despite of *PS* being chemically relatively inert, it does by far not show etch resistances as large as an inorganic hard mask would do. This leads to fast mask wear and requires exact knowledge about the film thickness of the initial block copolymer film to design a successful pattern transfer process. Nanometric fluctuations of the block copolymer thickness throughout the wafer/chip area due to microscopic impurities or other process-related issues may lead to difficulties in the pattern transfer process.

The self-assembly of block copolymers between two grain boundaries is quantized to multiples of 0.5 L_0_, because of the adjacent horizontally oriented grains’ quantized height. This concept is clarified in Figure A3, where we present a measurement of the block copolymer height in the horizontally aligned (and previously modified) area. The block copolymer has been removed by m-AFM in an area orthogonal to the long axis of the modified area, and an AFM height image of one part of the sample is depicted in Figure A3a. A single line AFM height profile is shown in Figure A3b. The profile’s precise location and direction is indicated by the white dashed line both in the sketch and in the actual height image. The step height is 35 nm, which corresponds to 1.5 L_0_ (with L_0_ being 23.4 nm). Based on the knowledge that the silicon wafer is preferentially wetted by *PMMA*, we conclude that the top layer must be *PS*, as sketched in Figure A3c.

Figure A3d presents a sketch of four different self-assembly heights in steps of 0.5 L_0_. We would like to state at this point that the horizontally aligned areas will self-assemble in a quantized state such that the film height in the horizontally aligned area is always an integer multiple of 0.5 L_0_. Interestingly, we observe in all our experiments that the self-assembly of block copolymers in the vertically aligned trapped grain is few nanometers recessed with respect to the horizontally aligned grains. The opposite has been observed by other authors in a similar experiment [58], where the vertically assembled block copolymer domain is few nanometers higher than the horizontally aligned domain. In both cases the deviation of the vertical lamellae height from the quantized horizontal lamellae height is few nanometers.

### Appendix A.3. Grain-Boundary-Induced Alignment with Ternary Blends

For the application of grain-boundary-induced alignment it could be favorable to achieve an alignment mode that is not restricted by the correlation length of the block copolymer. One way to do so is to influence the material is such a way that the preferentially formed grain boundary is a 90° tilt grain boundary (see Figure A1c) instead of a 90° twist grain boundary (see Figure A2b). This is the case when the vertical interface created by the horizontally ordered block copolymer is energetically more attractive to one block than for the other, similar to the energetic situation in graphoepitaxy.

Duque et al. [39] calculated the free energy in twist and tilt grain boundaries for a pure diblock copolymer and a ternary blend containing 70% diblock copolymer and a 30% fraction of homopolymers. The calculation yields that 90° tilt grain boundaries have the same (or in the concrete case of mixing in 30% homopolymers an almost negligible 2% lower) grain boundary free energy compared to the 90° twist grain boundary in case of mixing in homopolymers. The 90° twist grain boundary has, in turn, a clear energetic advantage over the 90° tilt grain boundary in case of pure diblock copolymers. Moreover, the absolute grain boundary free energy for the ternary blend is reduced heavily to approximately 1/3 of the value for the pure block copolymer.

We reproduced this experiment using a blend of the 23.4 nm pitch *PS-b-PMMA* and different amounts of *PS* and *PMMA* with a molecular weight of 39.5 kg/mol with a total accumulated homopolymer fractions between 0% and 45%. In all the experiments, the volume fraction of *PS* and *PMMA* is equal. Results of the behavior of the ternary block copolymer/homopolymer blend in the vicinity of a grain boundary is presented in Figure A4. The annotation in the respective SEM images in Figure A4 refers to the accumulated homopolymer fraction, which means that “30% HP” in Figure A4b indicates that the ternary blend contains 15% *PS*, 15% *PMMA,* and 70% *PS-b-PMMA*.

We observe two different effects caused by the increasing homopolymer fraction. At one hand, we observe a progressive swelling of the block copolymer features due to the accumulation of homopolymer molecules in the center of the domain (because this is the place where the molecules can avoid the energetically unfavorable location close to the IMDS). This effect is accompanied by a decrease of the material’s correlation length.

On the other hand, we observe that the proportion of tilt grain boundaries rises upon increasing the homopolymer ratio. Those parts of the grain boundary that correspond to a tilt grain boundary are red shaded, while twist grain boundaries are marked blue (see Figure A4). As a general trend we can state, that the block copolymer is more likely to form tilt grain boundaries at increasing homopolymer fractions. This behavior is explained by the reduction of the free energy difference between the two grain boundary morphologies. The formation of tilt grain boundaries does, however, not convert into the energetically clearly favorable state. The proportion of tilt grain boundaries and twist grain boundaries for the 45% homopolymer sample is approximately equal, as shown in Figure A4c. This observation indicates that the energetic difference between the two states is very low.

The desired situation we describe in the introductory paragraph is, however, only achieved if the formation of a tilt grain boundary is energetically favorable for one particular block, but not for the other one. This effect may be better understood when we consider ref. [59], where the authors observe that added *PS*-coated *Au* nanoparticles accumulate particularly in grain boundaries. There, the nanoparticles minimize the A/B interface area of the—in that case—*PS-b-PEO* block copolymer in favor of the *PS*-block. The alignment of block copolymers via grain-boundary-induced alignment parallel to the grain boundary can be successful, in case two prerequisites are fulfilled: (i) the total homopolymer fraction in the blend is large enough to lower the tilt grain boundary free energy significantly and (ii) the ratio of the two homopolymers in the blend is sufficiently asymmetric that the tilt grain boundary is reduced significantly more for one block than for the other.

In reference [60] the authors make use of asymmetric ternary blends with a total of 40% homopolymers (e.g., 29% *PMMA* and 11% *PS*) to provoke the formation of tilt grain boundaries. The alignment of the block copolymers in that work, is however accompanied by the use of high-resolution chemical guiding patterns, which we believe would not be necessary for sufficiently high-correlation-length blends. The effect of having worked with a ternary blend could represent an explanation for the unusual self-assembly morphology that is observed, but not further explained, in reference [58].
